# Early-stage effect of HIBD on neuro-motor function and organic composition of neurovascular units in neonatal rats

**DOI:** 10.3389/fnins.2023.1242936

**Published:** 2023-11-21

**Authors:** Yanjun Mo, Ying Zeng, Luyao Huo, Gang Liu, Jingwei Tao, Yu Jiang, Tuo Zhao, Zhuoluo Zhou, Xiaohong Mu

**Affiliations:** Dongzhimen Hospital, Beijing University of Chinese Medicine, Beijing, China

**Keywords:** hypoxic–ischemic brain damage, neuro-motor function, cerebral blood flow, microvasculature, neuron, microglia, astrocytes

## Abstract

**Objective:**

This study aimed to investigate the effects of neonatal hypoxic–ischemic brain damage (HIBD) on early-stage neuro-motor function, cerebral blood flow, and the neurovascular unit.

**Methods:**

Twenty-four Sprague–Dawley newborn rats aged 7 days were obtained and randomly assigned to either the sham or the model group using a random number table. The HIBD model was established using the Rice-Vannucci method. After the induction of HIBD, the body weight of the rats was measured and their neuro-motor function was assessed. Further, cerebral blood flow perfusion was evaluated using laser speckle flow imaging, and immunofluorescent staining techniques were employed for examining the activation of specific markers and their morphological changes in different cell populations, which included vascular endothelial cells, neurons, astrocytes, and microglia within the motor cortex.

**Results:**

After HIBD, the model group exhibited impaired neuro-motor function and growth. Cerebral blood flow perfusion decreased in both the hemispheres on day 1 and in the ipsilateral brain on day 4. However, no significant difference was observed between the two groups on day 7. Moreover, the CD31 and NeuN showed a sharp decline on day 1, which was followed by a gradual increase in the expression levels. The activated microglia and astrocytes formed clusters in the injured cortex. Notably, the regions with positive staining for Arg-1, Iba-1, CD68, and GFAP consistently displayed higher values in the model group as compared to that in the sham group. The total number of branch endpoints and microglia branches was higher in the model group than in the sham group. Immunofluorescent co-localization analysis revealed no co-staining between Iba-1 and Arg-1; however, the Pearson’s *R*-value for the co-localization of Iba-1 and CD68 was higher in the model group, which indicated an increasing trend of co-staining in the model group.

**Conclusion:**

Early-stage neuro-motor function, cerebral blood flow, microvasculature, and neurons in neonatal rats exhibited a trend of gradual recovery over time. The activation and upregulation of neuroglial cells continued persistently after HIBD. Furthermore, the impact of HIBD on early-stage neuro-motor function in newborn rats did not synchronize with the activation of neuroglial cells. The recovery of neuro-motor function, microvasculature, and neurons occurred earlier than that of neuroglial cells.

## Introduction

Hypoxic ischemic brain damage (HIBD) is caused by perinatal hypoxia-asphyxia, and it can result in neonatal hypoxic–ischemic encephalopathy (HIE) and subsequent cerebral palsy (CP). Prolonged hypoxia leads to decreased fetal cardiac output, which diminishes cerebral blood flow. The subsequent vascular insufficiency triggers a series of distinct neuropathological changes that lead to various clinically notable manifestations (Douglas-Escobar and Weiss, 2015; Massaro et al., 2018). However, the molecular mechanisms that underlie brain injury in infants with HIBD and the relevant effective treatment approaches remain unclear.

Insufficient cellular blood delivery and reduced cerebral oxygen levels initiate a cascade of enzymatic reactions in the cells, resulting in neuronal degeneration or necrosis as well as delayed neuronal cell differentiation and myelination. These processes are interconnected, and they collectively impact the intricate progression of brain development and maturation. Furthermore, prolonged hypoxia-ischemia(HI) may potentially cause irreversible damage to the brain and lead to long-lasting neurological consequences such as cognitive impairment and CP onset (Yang et al., 2020). CP etiology typically includes neonatal HIBD, maternal infections during pregnancy, intrauterine trauma, and preterm birth (Galea et al., 2019; Sanches et al., 2021). Notably, hypoxia-ischemia plays a crucial role in CP diagnosis (Kapanova et al., 2021). The Rice-Vannucci model is widely used in animal studies, particularly in rodent models of CP, wherein cerebral HI is induced to simulate CP (Cavarsan et al., 2019). Further research about the underlying mechanisms and development of effective treatment strategies for CP is required urgently. In the current study, the Rice-Vannucci model of induction was adopted to simulate CP by HI.

The conceptualization of the neurovascular unit (NVU) has evolved, which initially focused on the intricate interaction between the brain vasculature and neurons to encompass a biological unit comprised of brain microvasculature, neurons, and glial cells. Every component of this unit plays a distinct role in the maintenance of the stability of the structure and function of the central nervous system (Wang et al., 2021). Moreover, neurons are responsible for the transmission of nerve signals and processing information, and the microvasculature supply the neurons with nutrients and oxygen and regulate the local blood flow to meet the metabolic demands of the neurons. In conjunction with the glial cells, the microvasculature uphold homeostasis in the brain (Ahmad et al., 2020). NVU dysfunction occurs with HI injury to the brain, which changes the brain microenvironment and triggers a cascade of reactions. Currently, early alterations in the neurovascular unit in neonatal HIBD rats are not completely understood.

In the current study, an HIBD neonatal rat model was used that was established by ligating the left carotid artery, which subsequently induced hypoxia. During the early stages, day 1, 4, and 7 post-modeling, the neuro-motor function, cerebral blood flow, and NVU of the rats were examined. Using immunofluorescence, microvasculature (labeled with CD31), neurons (labeled with NeuN), activated astrocytes (labeled with GFAP), as well as activated microglia (labeled with Iba-1, Arg-1, and CD68) were specifically observed. The current research aimed to elucidate the changes induced by HI in the aforementioned components and to establish a foundation for the subsequent experiments.

## Materials and methods

This study received approval (No. IBTCMCACMS21-2304-01) on April 18, 2023, from the Animal Ethics Committee of the Institute of Basic Theory of Traditional Chinese Medicine, China Academy of Chinese Medical Sciences. The research adhered to the guidelines established for the care and use of laboratory animals by the National Institutes of Health (NIH), United States of America, as published by the National Academy Press, Washington, D.C.

### Animals

Two pregnant Sprague–Dawley (SD) rats at approximately 19 days of gestation were provided by SiPeiFu (Beijing) Biotechnology Co., Ltd., with license number scxk (Jing) 2019-0010. The pregnant rats were housed in a specific pathogen-free (SPF) animal facility at the Institute of Basic Theory of Traditional Chinese Medicine, China Academy of Chinese Medical Sciences. They had unrestricted access to food and water and were housed in a 12-h light–dark cycle (6 am to 6 pm). The room temperature was maintained at 20–26°C with a relative humidity of 55 ± 5.5%. The day of delivery was considered postnatal day 1 (P1).

**Figure 1:**
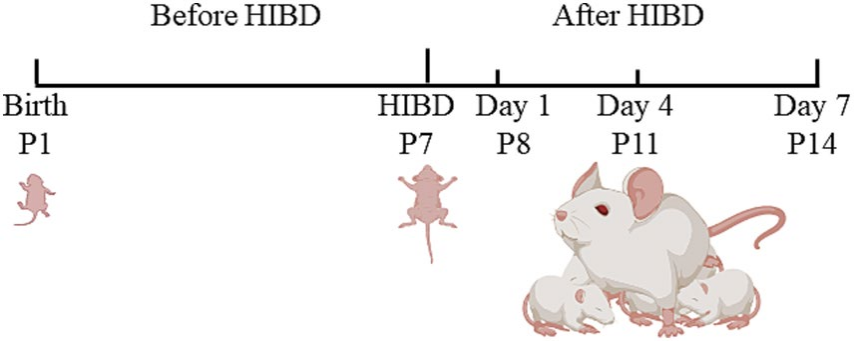
The schematic diagram of the experimental timeline. Neonatal rats underwent HIBD modeling on P7. The left common carotid artery was exposed and ligated at both ends, followed by a cut in the middle. Once the pups regained consciousness, they were placed in a sealed chamber and exposed to 2.5 h of hypoxic gas. Subsequently, they were returned to the cage for rearing. On day 1 (P8), day 4 (P11), and day 7 (P14), the rats underwent neurobehavioral assessments, laser speckle perfusion imaging, and brain tissue sampling.

We used neonatal SD rats at P7, with undifferentiated gender and weights ranging from 13 to 21 g. Each litter consisted of 12–18 pups. After modeling, the HIBD pups were randomly divided into three groups: day 1 (P8), day 4 (P11), and day 7 (P14). Additionally, a sham group was established as a control, with six pups included in each group. In the immunofluorescence staining section, Day 1, Day 4, and Day 7 refer to the model group at 1, 4, and 7 days post-modeling, respectively. Feeding pregnant rats until delivery enabled accurate determination of the birth date, facilitated maternal lactation, and minimized disturbances caused by pup transfers. Figure 1 presents a schematic diagram of the experimental timeline.

### Establishment the HIBD model

The classic Rice-Vannucci method was employed for modeling HIBD (Vannucci and Vannucci, 2005). On day 7, 7-day-old SPF SD rats were placed in an animal anesthesia machine (R620-S1, Revode, Shenzhen, China) induction chamber and administered vaporized isoflurane (R540, Revode, Shenzhen, China) for anesthesia. The rat’s limbs were secured to the operating table using adhesive tape, while low-concentration anesthesia was maintained using a mask (2.5% isoflurane for induction and 1.5% for maintenance). After sterilizing the neck skin with iodine, a 3 mm incision was made. Fine forceps were used to separate the left carotid artery and the surrounding tissues, and the exposed ends of the artery were ligated before it was cut in the middle. The surgical procedure was performed gently to minimize bleeding and completed within 8 min. The incision was sutured, the bloodstains were cleaned, and a medical liquid dressing was applied to the injury site. After recovery, the pups were placed in a custom-made airtight hypoxia chamber, wherein they were exposed to a gas mixture that consisted of the following volume fractions: 92% N_2_ and 8% O_2_. An oxygen monitor was placed in the chamber to maintain 8% of regulated oxygen concentration by adjusting the ventilation rate. Furthermore, the hypoxic chamber was wrapped in an electric blanket to maintain an internal temperature of 36°C. Excessively low temperatures should be crucially avoided, as they can alleviate HIBD (Allen and Brandon, 2011). After 2.5 h of hypoxia, the neonatal rats were removed from the chamber and returned to their cages for rearing. In the sham group, a midline incision was performed on the skin of the neck, and a suture was made without ligation of the carotid artery or hypoxia treatment.

### Determining the success of the modeling

The surgical procedure of HIBD was performed on the experimental group of rats. On day 1, the Zea-Longa scoring system was used to evaluate the extent of neurofunctional deficits in the HIBD rat model. The rats with Zea-Longa scores between 2 and 3 were included in the experiment, which indicated successful HIBD modeling. Only those rats that exhibited successful HIBD modeling were chosen for the subsequent experiments, and those that exhibited failed HIBD modeling were excluded. The sample size was 6 in both the model as well as the sham group (*n* = 6).

### Neural-motor function tests

On the first, fourth, and seventh day following the modeling procedure, both groups of rats underwent a series of tests, which included body weight assessment, forelimb suspension, hindlimb suspension, negative geotaxis, surface righting, and cliff aversion tests. The methodology for these tests can be found in the Feather-Schussler, D. N. paper and video (Feather-Schussler and Ferguson, 2016). The behavioral experiments will be conducted by a blinded researcher, unaware of the experimental grouping.

### Zea-Longa

Scoring criteria for the behavioral assessments were as follows: 1 score: No discernible changes in limb usage were observed; 2 score: Inability to fully extend the contralateral limb while under tail suspension; 3 score: Circling toward the contralateral side during tail suspension; 4 score: Leaning or falling toward the contralateral side while walking; and 5 score: Complete inability to walk spontaneously, possibly accompanied by seizures, unconsciousness, or loss of consciousness.

### Surface righting

The experimenter lifts the tail of rat and places it with its back facing downward on a horizontal board. The index finger of the right hand is placed against the chin of rat, while the thumb is placed against its hind limbs. The right-hand releases the two fingers, and at the same time, the left-hand starts the stopwatch to begin timing. If the rat flips over spontaneously within the first attempt, the time of the previous flip is recorded as the recovery time. Otherwise, if the rat fails to flip within the first attempt, the time of the second flip is recorded, and this time is considered as the recovery time.

Following each trial, a designated time interval is observed before repeating the process for a total of three data sets. If the rat maintains an abnormal posture and fails to actively return to the normal position, the presence of the righting reflex is considered absent. The righting reflex is an innate response whereby a juvenile rat can flip from a supine position to an upright position with its back facing upward, independent of learned behavior.

### Forelimb suspension

The forelimb suspension test assesses the forelimb strength of the rat, encompassing the arm and paw muscles. During the test, the rat grasps a thin iron wire attached to an uncovered large beaker, ensuring that the hind limbs do not touch the inner wall of the beaker when the rat twists its body. To maintain stability, a piece of cotton is positioned at the bottom of the beaker. The duration it takes for the rat to release its grip from the wire, indicating the inability to sustain support with its forelimbs, is recorded. The dropout times from both groups are then compared for analysis.

### Hindlimb suspension

This experiment is designed for neonatal rats aged up to postnatal day 14. A typical 250 mL cylindrical beaker, equipped with a cushioning cotton pad at the base, is utilized. Carefully grasping the rat’s tail, the experimenter positions it head-down into the beaker, ensuring that its hind limbs rest on the rim. Subsequently, the experimenter releases the rat’s tail and measures the time it takes for the rat to descend to the bottom of the beaker.

### Negative geotaxis

The purpose of this test is to evaluate the motor coordination of the rat. It involves securing the rat in an inverted position, with its head facing downwards, on a 45° inclined plane for a duration of 5 s. The time taken by the rat to complete a 180° rotation and position its head upwards after being released is noted. Three repetitions of the experiment are conducted for each rat, and the average value is calculated. In cases where rats fall off the inclined plane or fail to complete the rotation, they can be retested. If a rat rolls down the slope due to exhaustion, it is given a rest period before the retest.

### Cliff aversion

The Cliff Aversion test is conducted to evaluate maze reflexes, strength, and coordination in rats ranging from postnatal day 1 to 14. A cardboard box containing a flat elevated platform is utilized, and the rat is positioned at the edge, with only its front paws and nose making contact with the edge. Subsequently, the time taken for the rat to turn away from the cliff and retract its paws and nose from the edge is recorded. If the rat falls off the edge, an additional trial can be conducted. Once the rat has successfully moved both its nose and paws away from the edge and completed the turn, the timing is stopped, and the time is recorded. The test is repeated three times.

It is important not to conduct the test when the rat is fatigued. Sufficient rest should be provided to the rat after each trial to allow for energy recovery and minimize potential interference with the test results.

### Cerebral blood flow detection

#### Laser speckle blood flow imaging

Anesthesia was administered using a 1.25% concentration solution of tribromoethanol. Once the animals were adequately anesthetized via intraperitoneal injection, they were secured onto a stereotaxic instrument. The rats were positioned in a prone posture, and the skin over their heads was prepared and sterilized. Subsequently, an incision was made in the scalp to expose the underlying subcutaneous tissue. To ensure a clean surgical site, the surface of the skull was gently wiped with a cotton swab soaked in 3% hydrogen peroxide. The laser speckle perfusion imaging system (PeriCam PSI Z, Perimed, Sweden) was carefully placed on the exposed skull, and the focal length was adjusted to measure blood flow on both sides of the rat’s brain. Laser speckle contrast images and average perfusion values were recorded for each experimental group, allowing for the analysis of changes in blood flow and imaging characteristics during the monitoring period. The sample size for both the model group and the sham group consisted of four rats (*n* = 4).

#### Irrigation and sampling

On the day of modeling the weight of the two groups of rats was measured. On days 1, 4, and 7 after modeling, behavioral tests were performed on both groups of rats, followed by laser speckle brain perfusion monitoring. After the observation period, the rats were immediately fixed in a supine position on the animal surgery table. They were over-anesthetized with a 1% concentration solution of sodium pentobarbital. A reverse “T” incision was made to open the thoracic and abdominal cavities, taking care to protect the arteries, heart, lungs, and liver. The diaphragm and ribs were cut open to fully expose the heart. Using a syringe needle, the left ventricle near the apex of the heart was punctured and inserted obliquely into the ascending aorta, being careful not to puncture the aorta or the heart. Hemostatic forceps were used to secure the syringe needle and the surrounding skin of the incision, providing adequate exposure to the surgical field. The right atrium was incised using ophthalmic scissors to create open body circulation, and approximately 50 mL of physiological saline was rapidly infused, starting with a fast flow and then slowing down, to drain the blood from the rat’s body. The liver and lungs turned white, and the infusion was stopped when the fluid became transparent. Following this, approximately 50 mL of 4% paraformaldehyde, pre-cooled to 4°C, was continuously infused to achieve fixation, resulting in the rat’s liver, tail, and entire body becoming rigid and stiff. Next, decapitation was performed, and the posterior scalp of the rat was peeled back to carefully separate the skull using scissors. The intact brain tissue was carefully removed and placed in a 4% paraformaldehyde solution for fixation for 2 h, followed by transfer to a 30% sucrose solution for dehydration.

#### Immunofluorescence

Neurons, microvasculature, astrocytes, and microglia play important roles as components of the neurovascular unit, and observing their morphology can help determine their function. The sample size in both the model group (day 1, day 4, and day 7) and the sham group was 6 (*n* = 6). The brains were obtained by perfusing 4% paraformaldehyde through the rat heart, followed by dehydration in a 30% sucrose solution for 24 h. The brains were then placed in a flat freezing microtome (REM-710, Yamato Koki Industrial, Japan) and cut into 80 μm thick sections, which were subsequently stained. Blocking: The sections were removed and blocked with a solution containing 3% serum, 1% Triton X-100, and 0.1 M phosphate buffer (PB; pH 7.4) for 1.5 h. After incubation, the blocking solution was discarded. Primary antibody incubation: Corresponding antibodies were selected and incubated with the sections overnight at 4°C. Washing: The sections were washed three times on a shaker with 0.1 M PB solution for 5 min each. Secondary antibody incubation: Corresponding secondary antibodies were selected and incubated at room temperature in the dark for 2 h. Washing: The sections were washed three times with 0.1 M PB for 5 min each. Mounting: The tissues were spread out on positively charged glass slides using a brush, and an appropriate amount of 50% glycerol was added as a mounting medium. The tissue was observed using a fully automated panoramic tissue cell scanning analysis system (VS120, Olympus, Japan). The results of immunostaining in the rat motor cortex were photographed and subjected to qualitative and semi-quantitative analysis using a multiphoton spectral confocal microscope (FV1200, Olympus, Japan), with excitation wavelengths of 405, 488, and 594 nm. Image processing and annotation were performed using Adobe Photoshop CC 2017, Adobe Illustrator CC 2017, ImageJ 1.53 t, and its plugins, which included positive cell area ratio, cell skeletonization processing, and immunofluorescence colocalization analysis. Primary antibody sources and concentrations: CD31 for labeling endothelial cells (1:500, AF3628, R&D Systems, United States), NeuN for labeling neurons (1:1,000, ab104224, Abcam, United Kingdom), GFAP for labeling astrocytes (1:1,000, G3893, Sigma, United States), Arg-1 (1:50, ab60176, Abcam, United Kingdom), Iba-1 (1:1,000, ab178847, Abcam, United Kingdom), and CD68 (1:1,000, MA5-16654, Thermo Fisher, United States).

### Statistical analysis

Statistical analyses were performed using SAS 9.2. All the data were metrological and expressed as mean ± SEM. T-test and Wilcoxon two-sample test were used to compare the differences between the two groups. The results were considered statistically significant at *p < 0.05*.

## Results

### Modeling situation

On day 1 after modeling, we performed Zea-Longa scoring to evaluate the success of the model. Rats that exhibited scores ranging from 2 to 3 were assigned to the model group, whereas the sham group exhibited a score of 0. Unfortunately, during the modeling process, two rats died, which was likely attributed to prolonged the duration of the surgery and other factors. However, this loss was compensated for by acquiring additional rats as replacements. Throughout the subsequent experiments, no further instances of mortality occurred.

### Impact of HIBD on neuro-motor function

#### Body weight

**Figure 2:**
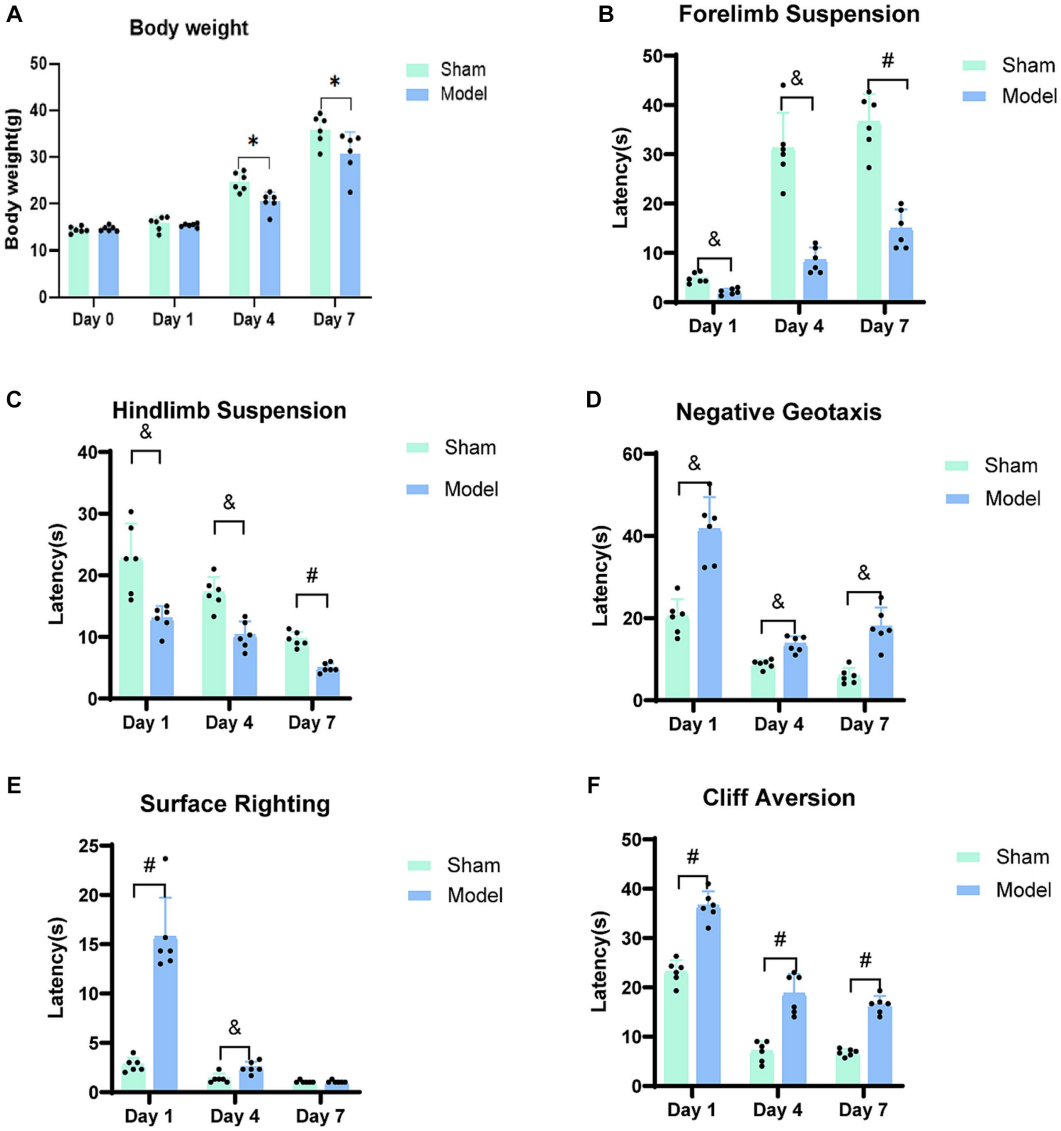
Neuro-motor function bar graph. **(A)** The body weight of rats on the day of modeling (Day 0) and at three-time points. **(B)** Forelimb Suspension. **(C)** Hindlimb Suspension. **(D)** Negative Geotaxis. **(E)** Surface Righting. **(F)** Cliff Aversion. A progressive restoration of neuro-motor function was observed in rats with HIBD over the course of the study period. ^*^p < 0.05, ^&^*p < 0.01*, ^#^*p < 0.0001* (vs. sham group).

No significant differences between the model and sham groups (*p = 0.5007*) were observed regarding baseline body weights, which indicated consistent initial weights. Similarly, no significant disparities in body weights were observed on the first day after modeling (*p = 0.5501*). However, on days 4 (*p = 0.0041*) and 7 (*p = 0.0487*), the sham group exhibited higher body weights as compared to the model group (Figure 2A). Regarding growth and development, neither group displayed eye-opening or ear-spreading on days 1 and 4 post-modeling. By day 7, some rats in the model group displayed the situation of one eye open and one closed, while the majority of the rats sham group displayed fully opened eyes. Notably, the sham group demonstrated superior developmental progress with eye-opening and ear-spreading as compared to the model group, which indicated delayed developmental advancements in rats subjected to the modeling procedure.

#### Forelimb suspension

The duration of forelimb suspension in the HIBD model group rats decreased as compared to that in the sham group (Figure 2B). Significant differences were observed between the two groups on days 1 (*p = 0.0003*) and 4 (*p = 0.0003*) post-modeling, and a further significant difference was observed on day 7 (*p < 0.0001*). Both the model and sham groups exhibited an increased duration of forelimb suspension, which was potentially attributed to the progressive maturation in the rats. However, the average duration of suspension in the model group was approximately 50% of that in the sham group, which suggests weaker forelimb strength in the model rats, influenced by the adverse effects of HIBD.

#### Hindlimb suspension

The hindlimb strength and neuromuscular function of the model group were weaker as compared to those of the sham group on days 1 (*p = 0.0068*), 4 (*p = 0.0006*), and 7 (*p < 0.0001*; Figure 2C). Notably, this test was similar to the forelimb suspension test and can be learned, particularly when cotton was placed at the bottom of the beaker to offer protection from the fall and significantly reduce the perception of pain for the rats due to the limited height of the beaker. Consequently, the overall duration of suspension displayed a declining trend, which could be attributed to the lack of cooperation by the rats during the experiment.

#### Negative geotaxis

As compared to the sham group, the model group showed significantly prolonged latency for head-turning on an inclined slope on days 1 (*p = 0.0002*), 4 (*p = 0.0003*), and 7 (*p = 0.0002*; Figure 2D). Upon analyzing the overall trend, it was observed that both groups exhibited a gradual decrease in the time taken for head-turning, which indicated improved motor coordination due to enhanced growth and development. Remarkably, the model group displayed signs of recovery in their ability of motor coordination.

#### Surface righting

The rodent species exhibit the righting reflex at an average age of approximately 5 days after birth. In the current experiment, the rats were included from day 7 after birth, indicating that they had developed surface righting. Compared to the sham group, the model group took longer to regain the backup position on day 1 (*p < 0.0001*) and 4 (*p = 0.0055*) after modeling; however, there were no significant differences between the two groups on day 7 (*p = 0.2974*; Figure 2E). Generally, the time taken for surface righting by both the groups decreased with time, and a sharp decline was observed in the taken for surface righting for the model group on day 4.

#### Cliff aversion

The model group exhibited significantly prolonged latency at three time points in leaving the cliff edge, as compared to the sham group (*p < 0.0001*; Figure 2F). Longitudinal analysis of these time points revealed a decreasing trend in the time taken by both groups to leave the cliff. However, the model group consistently required more time as compared to the sham group for leaving the cliff edge, indicating persistent impairment in their response to leave the cliff edge.

#### Laser speckle blood flow imaging

**Figure 3:**
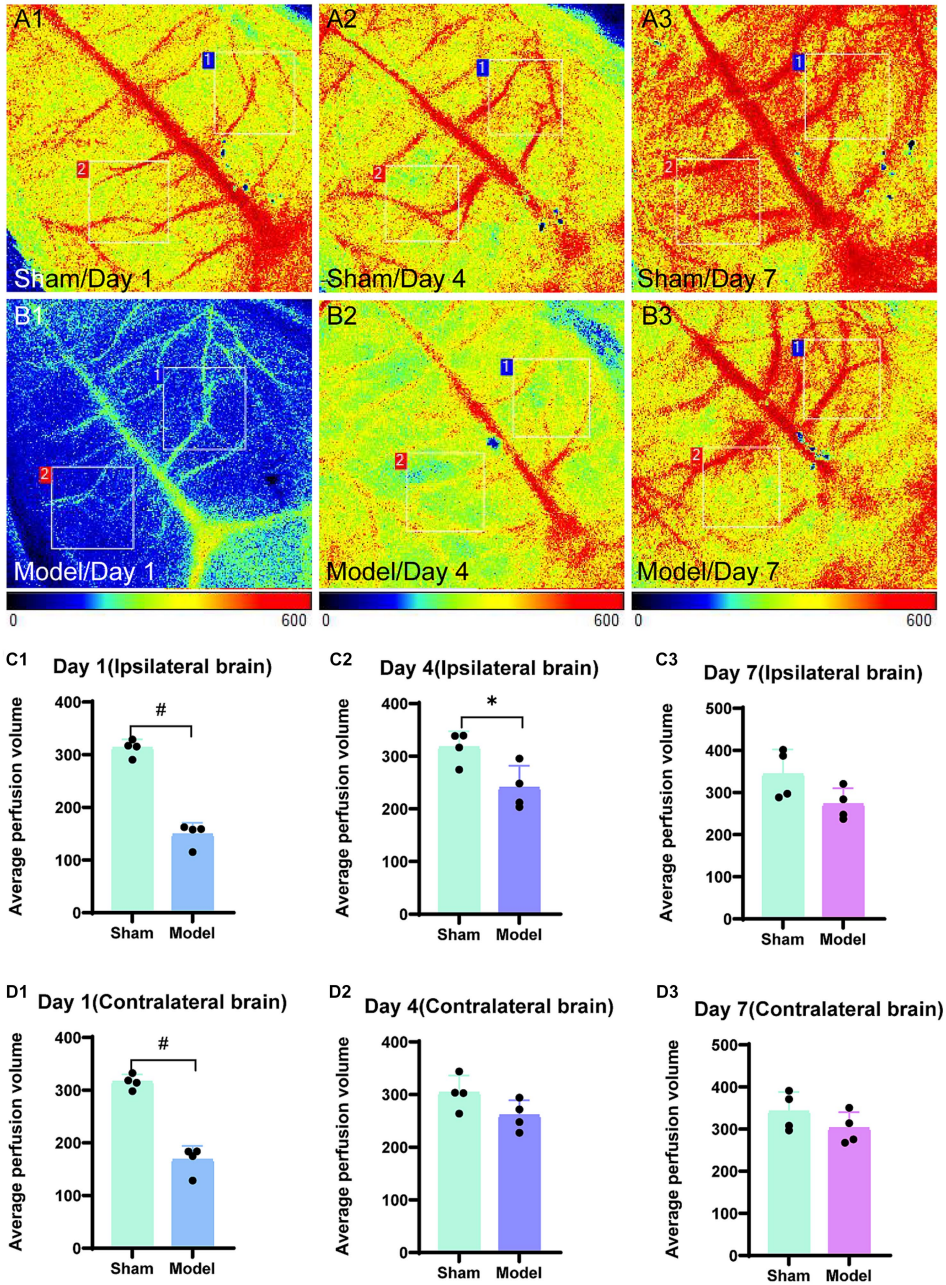
Laser speckle images of blood flow in the brain and average cerebral blood perfusion were assessed. Regions of interest (1 cm × 1 cm) were selected in the bilateral brain, specifically targeting corresponding areas on both sides. Region 1 was used to outline the ipsilateral brain and region 2 represented the contralateral brain. The average perfusion of cerebral blood was calculated within these regions, with a color bar scale ranging from 0 to 600. Representative images for the sham group are displayed in panels **(A1–A3)**, while those for the model group are depicted in panels **(B1–B3)**. The bar graphs are illustrating the average cerebral blood perfusion in the ipsilateral brain at three time points and are presented in panels **(C1–C3)**. Panels **(D1–D3)** represent the bar graphs for the contralateral brain. ^*^p < 0.05, ^&^*p < 0.01*, and ^#^*p < 0.0001* (vs. sham group).

In the sham group (*n* = 4), the blood flow of the brain was not significantly affected at the three time points following bilateral brain modeling, as observed from the laser speckle images (Figures 3A1−A3). On day 1, the model group (*n* = 4) exhibited blue laser speckle images, indicating reduced cerebral blood perfusion. Additionally, the ipsilateral brain showed a significantly shorter extension of cerebral blood flow as compared to the contralateral brain. On day 4, the degree of cerebral blood flow richness in the model group was lower in the ipsilateral brain as compared to that in the contralateral brain. On day 7, no statistically significant disparities were observed in the cerebral blood flow between the hemispheres of the brain in the model group (Figures 3B1−B3). According to the statistical data (Figures 3C1−D3), significant differences were observed on day 1 in the cerebral blood flow between the ipsilateral (*p < 0.0001*) and the contralateral brain (*p < 0.0001*) in the model group; moreover, the ligation of the left common carotid artery rapidly reduced the cerebral blood flow on both the ipsilateral and the contralateral sides. On day 4, the average perfusion of cerebral blood flow started increasing; however, it remained lower in the model group as compared to that in the sham group. A significant difference was observed between the two groups regarding cerebral blood flow for the ipsilateral brain (*p = 0.0242*), while no significant difference was observed between the two groups for the contralateral brain (*p = 0.0953*). On day 7, no statistically significant difference was noted in the cerebral blood flow between the ipsilateral (*p = 0.0886*) and the contralateral brain (*p = 0.2353*) for the model group as compared to the sham group, and the average cerebral blood perfusion was similar between the two groups.

### Brain tissue immunofluorescence

#### The impact of HIBD on microvascular endothelial cells

**Figure 4:**
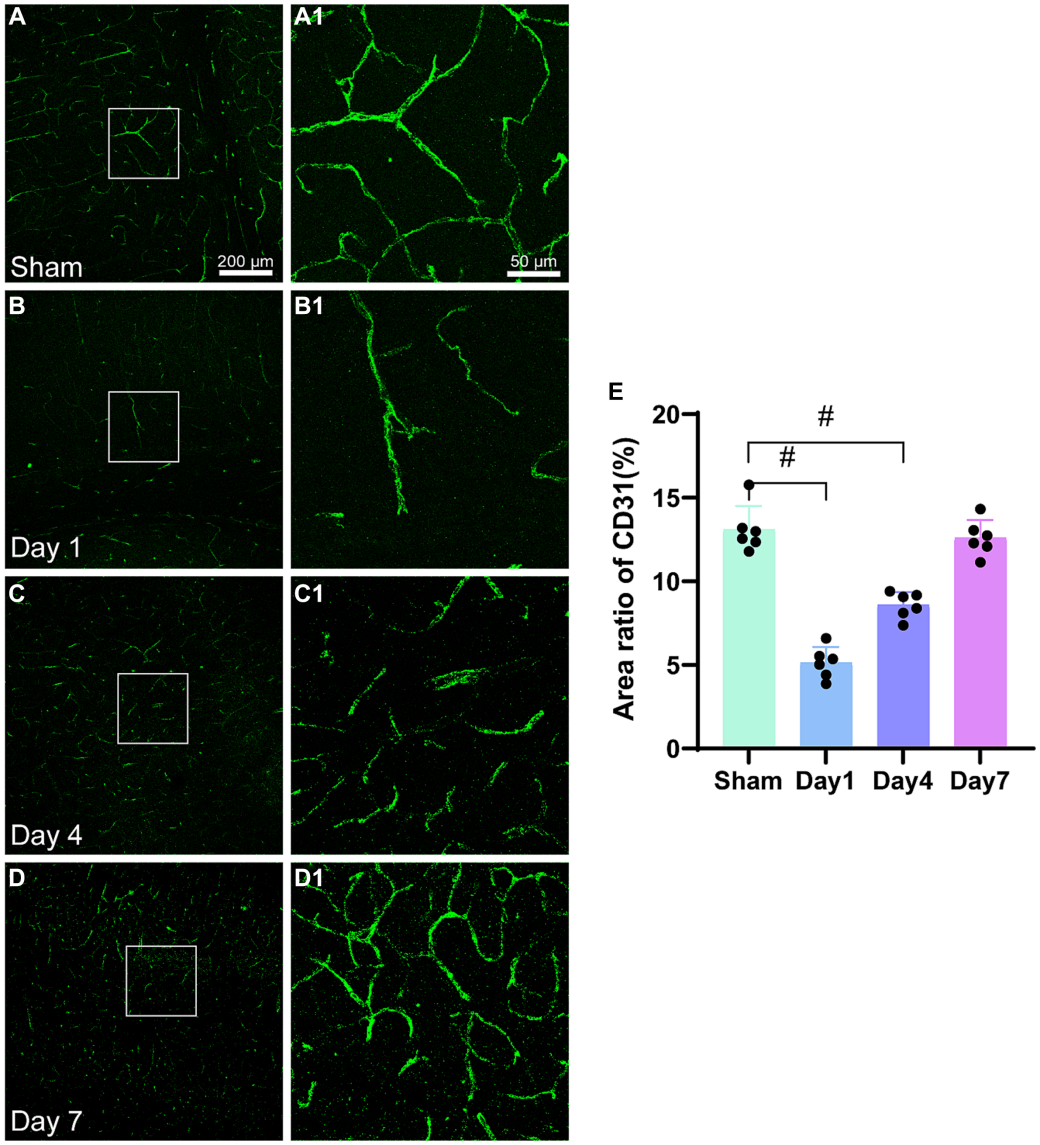
Representative images of CD31(green) in the cerebral cortex and the area ratio of CD31-positive cells. **(A–D)** Scale bar: 200 μm, **(A1–D3)** Scale bar: 50 μm. **(A,A1)** Representative images of the sham group; **(B,B1)** Representative images 1 day after modeling; **(C,C1)** Representative images 4 days after modeling; **(D,D1)** Representative images 7 days after modeling. **(E)** The bar graph shows the ratio of CD31-positive cell area to the total image area. ^*^p < 0.05, ^&^*p* < 0.01, ^#^*p* < 0.0001 (vs. sham group).

CD31 is a marker for microvascular endothelial cells (Figures 4A–D). The examination of images obtained from immunofluorescence (Figures 4A1−D1) revealed damage to the cerebral cortex microvasculature after HI. Initially, the microvasculature density was low on day 1; however, it gradually increased on days 4 and 7. By day 7, the microvasculature density in the model group was similar to that in the sham group. The statistical analysis (Figure 4E) demonstrated a significantly lower area ratio of CD31-positive cells on day 1 (*p < 0.0001*) and day 4 (*p < 0.0001*) compared to the sham group. However, on day 7, no statistically significant difference were observed in the CD31 area ratio (*p = 0.4955*) between the model and sham groups. The expression level of CD31 showed a progressive increase in the model group; moreover, it reached a comparable area ratio as that of the sham group by day 7 post-modeling.

#### The impact of HIBD on neurons and astrocytes

**Figure 5:**
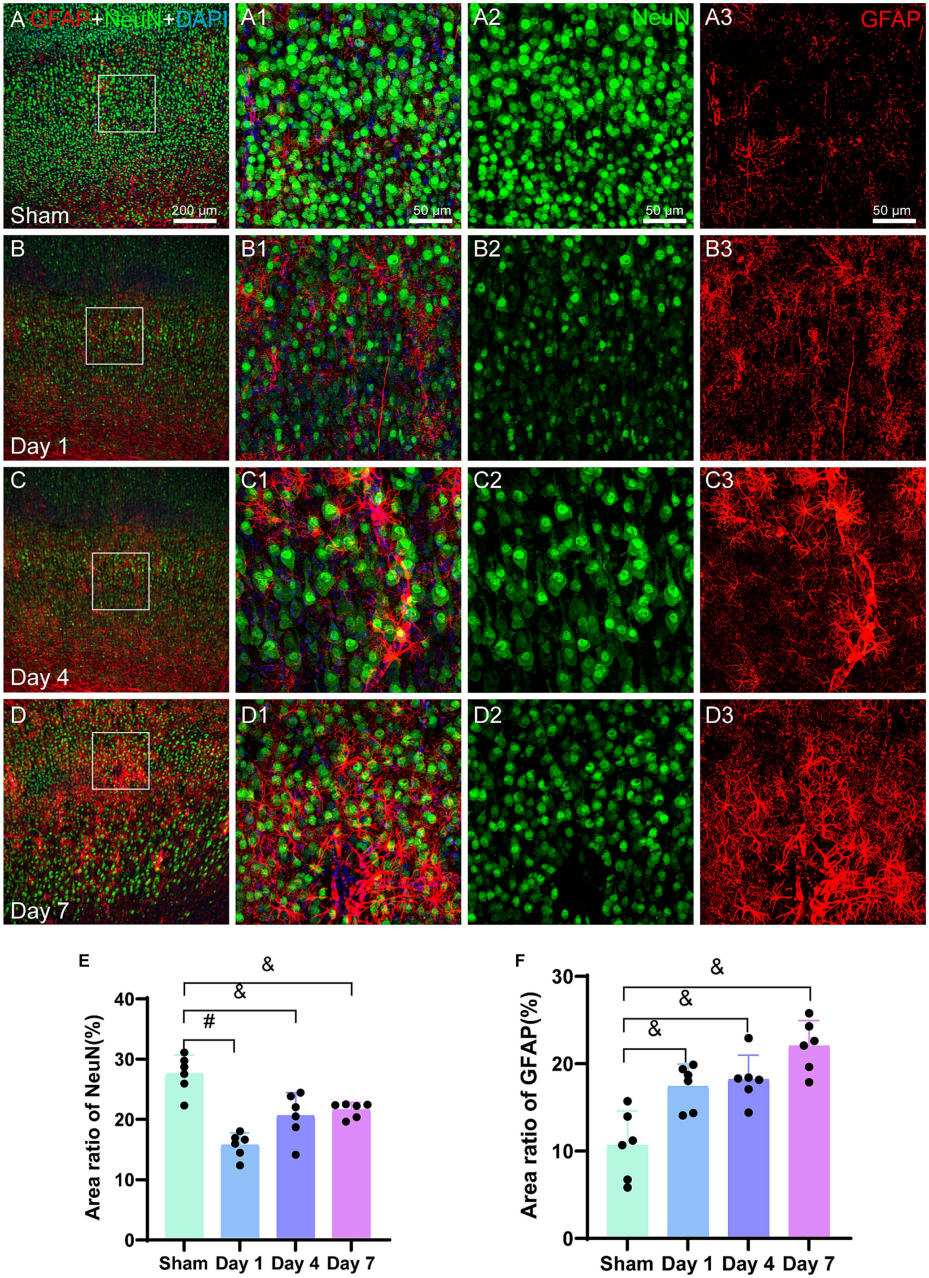
Representative images of NeuN (green) and GFAP (red) in the cerebral cortex. The bar graphs show the area ratio of the positive cells. **(A–D)** Scale bar: 200 μm, **(A1–D3)** Scale bar: 50 μm. **(A,A3)** A representative image of the sham group, where the distribution of NeuN is uniform and GFAP is in a resting state. **(B,B3)** The representative image of day 1 illustrates a decrease in the NeuN density along with an uneven distribution, indicating the activation of GFAP-labeled astrocytes. **(C,C3)** The representative image for day 4 reveals further activation of GFAP, which is characterized by an increase in the size of the cell body and thicker processes. **(D,D3)** The representative image for day 7 demonstrates a noticeable increase in the GFAP density, with a slight increase in the NeuN density. **(E)** The bar graph represents the ratio of the NeuN-positive cell area to the total image area. The ratios for day 1, day 4, and day 7 for the model group are lower than those for the sham group. **(F)** Similarly, a bar graph represents the ratio of GFAP-positive cell area to the total image area. The ratios for days 1, 4, and 7 are higher for the model group than those for the sham group. ^*^p < 0.05, ^&^*p < 0.01*, ^#^*p < 0.0001* (vs. sham group).

NeuN is a neuron-specific nuclear protein that is expressed in mature pyramidal and granule neurons in normal brain tissue. The analysis of immunofluorescence images (Figures 5A2−D2) revealed a sharp decrease in the NeuN density on day 1. Although the cell density of NeuN in the model group remained lower than that of the sham group on days 4 and 7, it had increased as compared to that on day 1. The bar graph (Figure 5E) shows a significant decrease in the area of NeuN-positive cells on day 1 in the model group as compared to that in the sham group (*p < 0.0001*). Furthermore, the area of NeuN-positive cells remained lower on days 4 (*p = 0.0063*) and 7 (*p = 0.0015*) for the model group as compared to the sham group. The area ratio substantially increased from day 1 to 4; however, this increase was not significantly different.

Glial fibrillary acidic protein (GFAP) serves as a marker for the identification of activated astrocytes. The immunofluorescence images in the current study (Figures 5A3−D3) revealed an increased density of GFAP-positive cells in the model rats during the early stages following HIBD. By day 4, the activated and migrated astrocytes were observed that were marked by GFAP. On day 7, further astrocyte activation was observed. The bar graph (Figure 5F) demonstrated a significant increase in the area ratio of GFAP-positive cells on days 1 (*p = 0.0053*), 4 (*p = 0.0031*), and 7 (*p = 0.0002*) for the model group as compared to the sham group, indicating a higher number of activated astrocytes in the former. Moreover, in the vicinity of damaged microvasculature, the area ratio on day 7 for the model group was twice as high as that for the sham group.

#### The impact of HIBD on microglia markers Iba-1 and Arg-1

**Figure 6:**
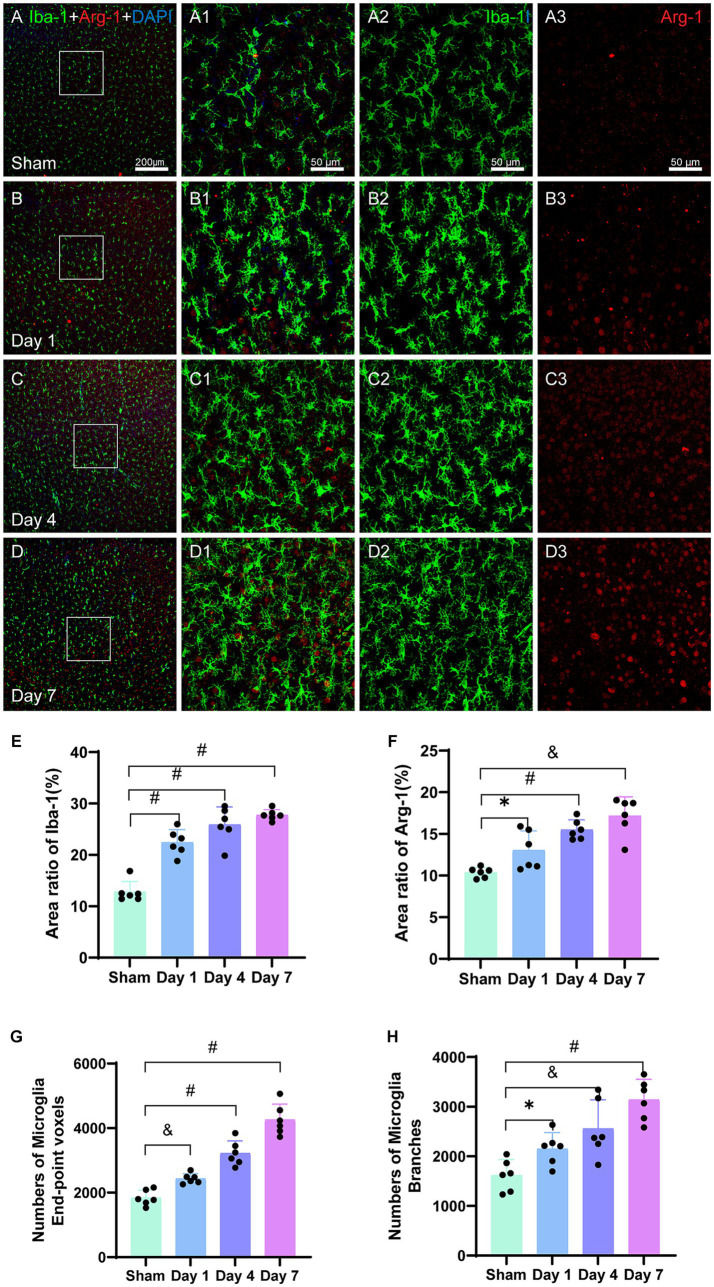
Representative images of Iba-1 (green) and Arg-1 (red) in the cerebral cortex. The bar graphs show the area ratios of positive cells. **(A–D)** Scale bar: 200 μm, **(A1–D3)** Scale bar: 50 μm. **(A–A3)** Representative image of the sham group showing evenly distributed Iba-1-labeled microglia and Arg-1. **(B–B3)** Representative image of activated microglia on day 1, with branched structures aggregating in the injured cortex. **(C–C3)** Representative image showing further activation and aggregation of microglia on day 4. **(D–D3)** Representative image showing Arg-1 surrounding Iba-1-labeled microglia on FIGURE 6 (Continued)day 7. **(E)** The area ratio of Iba-1-positive cells to the entire image area, with significant differences on days 1, 4, and 7 in the model group as compared to that in the sham group; further, the ratios were higher than that in the sham group. **(F)** The area ratio of Arg-1-positive cells to the entire image area, with differences on days 1, 4, and 7 in the model group as compared to that in the sham group; further, the ratios were higher than that in the sham group. **(G)** The total number of end-point voxels in microglia shows a continuous increase. **(H)** The total number of branches in the microglia shows a continuous increase. ^*^p < 0.05, ^&^*p < 0.01*, and ^#^*p < 0.0001* (vs. sham group).

Iba-1 is specifically expressed in the microglia, which are the only cells in the brain that express Iba-1. Analysis of the immunofluorescence images (Figures 6A2−D2) revealed the morphological characteristics of microglia in the sham group, including smaller cell bodies, longer processes, and fewer branches, which collectively indicated a resting state. However, on day 1, the activated microglia labeled with Iba-1 showed increased branching in the injured cerebral cortex. The activation and accumulation of microglia with highly branched structures continued in the motor cortex on days 4 and 7, leading to a gradual decrease in the proportion of the resting microglia. Analysis of the bar graph (Figure 6E) revealed that the area ratio of the Iba-1 positive cells was significantly higher for the model group on days 1 (*p < 0.0001*), 4 (*p < 0.0001*), and 7 (*p < 0.0001*) as compared to that for the sham group. Furthermore, the area ratio of the Iba-1 positive cells progressively increased over the experimental group. Notably, a rapid increase was observed in the Iba-1 positive cell area ratio after the first day of modeling.

The number of end-point voxels in the microglia was quantified (Figure 6G), and they were higher on day 1 in the model group as compared to that in the sham group (*p = 0.0005*). Similarly, significantly higher numbers of end-point voxels were observed on days 4 (*p < 0.0001*) and 7 (*p < 0.0001*) for the model group as compared to that in the sham group. Additionally, the total number of branches in the microglia was calculated (Figure 6H), which revealed a higher number on day 1 in the model group as compared to that in the sham group (*p = 0.0156*). Furthermore, day 4 had a significantly higher number of branches in the model group as compared to that in the sham group (*p = 0.0049*); furthermore, a substantial increase was observed on day 7 in the model group as compared to that in the sham group (*p < 0.0001*).

Arg-1 belongs to the arginase enzyme family, and it is expressed in various cell types such as microglia, red blood cells, liver cells, neutrophils, smooth muscles, and macrophages. In the current study, we investigated the expression of Arg-1 in the cerebral cortex, which is a pro-repair and alternative activation molecule. The analysis of the immunofluorescence images (Figures 6B1−D1) revealed a gradual increase in the density of Arg-1 surrounding Iba-1 in the injured ipsilateral cerebral cortex at all the three time points. Statistical analysis (Figure 6F) demonstrated that the area ratio of Arg-1 positive cells in the model group was significantly higher than that in the sham group on days 1 (*p = 0.0131*), 4 (*p < 0.0001*), and 7 (*p = 0.0051*). Arg-1 expression showed an early increase within 24 h of neonatal HIBD, and it continued to increase until day 7 post-modeling (Figures 6A3−D3).

#### The impact of HIBD on microglia markers Iba-1 and CD68

**Figure 7:**
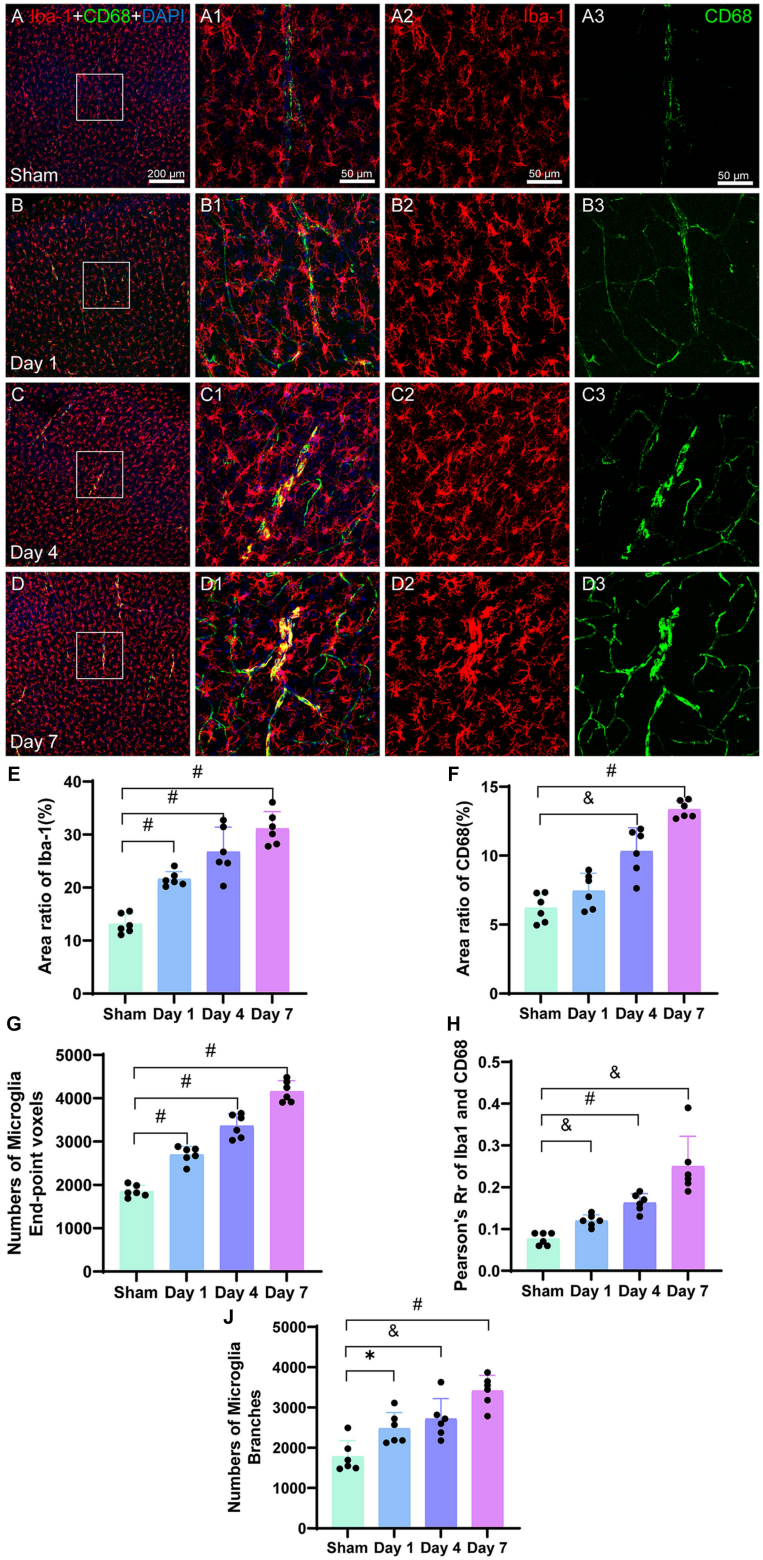
Representative images of Iba-1 (red) and CD68 (green) in the cerebral cortex. The corresponding bar graphs of the positive cell area ratio are shown. The scale bar for panels **(A–D)** is 200 μm and for panels **(A1–D3)** is 50 μm. Panels **(A–A3)** represent the sham image, wherein Iba-1-labeled microglia and CD68 are evenly distributed and have lower densities. Panels **(B–B3)** represent the image on day 1. Panels **(C–C3)** represent the image on day 4,FIGURE 7 (Continued)demonstrating the co-labeling of CD68 and Iba-1. Panels **(D–D3)** represent the image on day 7, demonstrating increased co-staining of CD68 and Iba-1. **(E)** The ratio of the Iba-1-positive cell area to the entire image area is presented, with differences between the model and sham groups at the three time points. The values of the model group were higher than those of the sham group. **(F)** The ratio of the CD68-positive cell area to the entire image area is presented, with differences between day 4, day 7, and the sham group. The values were higher than those of the sham group. **(G)** The number of microglia end-point voxels significantly increase and remain elevated post-HIBD in the mode group as compared to that in the sham group. **(H)** Analysis of fluorescent colocalization was performed for Iba-1 and CD68, with an increase in Pearson’s R of Iba-1 and CD68. **(J)** The activated microglia labeled with Iba-1 exhibit a highly branched morphology, with a higher count of total branches in the model group as compared to that in the sham group. ^*^p < 0.05, ^&^*p < 0.01*, and ^#^*p < 0.0001* (vs. sham group).

The immunofluorescence images of microglia labeled with Iba-1 (Figures 7A2–D2), revealed a gradual increase in the density of microglia in the model group, and aggregation was evident in the injured cerebral motor cortex (Figures 7A–D). Contrastingly, the microglia in the sham group appeared to be in a resting state (Figure 7A2). Following HIBD modeling, the microglia were activated, as evidenced by the increase in high number of microglia branching (Figures 7C1,D1). The area ratio of the positive microglia showed a progressive increase after modeling, with significant differences observed on days 1 (*p < 0.0001*), 4 (*p < 0.0001*), and 7 (*p < 0.0001*) in the model group as compared to the sham group (Figure 7E). We also quantified the number of end-point voxels in the microglia (Figure 7G) and found significantly higher counts on days 1 (*p < 0.0001*), 4 (*p < 0.0001*), and 7 (*p < 0.0001*) in the model group as compared to that in the sham group. Furthermore, we analyzed the total number of branches in the microglia (Figure 7J) and observed that the number of branches was higher on day 1 (*p = 0.0117*) in the model group as compared to that in the sham group. On day 4 (*p = 0.0049*), the number of branches was also higher in the model than in the sham group, and on day 7 (*p < 0.0001*), it branch number was significantly elevated in the model as compared to the sham group.

CD68 is a cytoplasmic glycoprotein associated with lysosomes that exhibits increased expression, which is linked to enhanced phagocytic function in cells. Immunofluorescence images (Figures 7A3−D3) illustrate a line-like morphology in the co-staining of CD68 with Iba-1 on days 4 and 7, potentially indicating the accumulation of CD68 around microvasculature injured by ischemia, which is associated with phagocytic functions. In the bar graph (Figure 7F), the CD68-positive cell area in the sham group is relatively low as compared to the model group. Following modeling, there was a slight increase in the area ratio of CD68 on day 1; however, this difference was not statistically significant as compared to that in the sham group (*p = 0.0929*). However, on days 4 (*p < 0.0001*) and 7 (*p < 0.0001*), the area ratio of CD68 was significantly higher than that in the sham group. The area ratio of CD68 continued to increase during the early stages after modeling.

The analysis of fluorescence co-localization between Iba-1 and CD68 was performed, and Pearson’s *R*-value was calculated. The results demonstrated minimal co-localization between Iba-1 and CD68 in the sham group. However, on days 1, 4, and 7, a gradual increase was observed in the co-localization between Iba-1 and CD68 in the microglia (Figures 7B1–D1). The co-localization on day 1 was significantly higher in the model group than that in the sham group (*p = 0.0004*). On days 4 (*p < 0.0001*) and 7 (*p = 0.0047*), the co-localization remained significantly higher than that in the sham group (Figure 7H). These findings suggested an augmented overlap and interaction between Iba-1 and CD68 in the activated microglia following HIBD modeling.

## Discussion

Appropriate animal models are essential to investigate the potential pathophysiological mechanisms and therapeutic targets in neonatal HIBD. In the current study, we utilized 7-day-old neonatal SD rats to establish the HIBD model, given that their brain histology is similar to that of human fetuses or newborns at 32–34 weeks of gestation. The HIBD model established by Vannucci and Rice is one of the most reliable animal models for simulating perinatal HIBD (Lan et al., 2023). Previous studies have elucidated that an injury occurs when homeostasis cannot be maintained by neurons and glial cells due to a prolonged lack of essential substrates, including oxygen and glucose (Gunn and Thoresen, 2019). The HIBD model induces permanent ischemia in the brain region that is supplied by the left common carotid artery, which results in the disruption of the cerebral microenvironment. Hypoxia is maintained for 2.5 h, and it reportedly causes significant damage to various brain regions such as the cortex, striatum, and hippocampus as revealed by magnetic resonance imaging and histopathological assessments (Rumajogee et al., 2016; Gonzalez Fuentes et al., 2021). The primary objective of our study was to observe the neurobehavioral function and changes that occur in the motor cortex of neonatal rats with HIBD. Notably, the blood flow in the contralateral hemisphere in this model was minimally affected by the occlusion of the common carotid artery. However, the contralateral hemisphere was affected by hypoxia; hence, it could not be considered as the control. To address this, a sham group was used as the control for the current study.

Heterogeneity in disease etiology prevents a single animal model from fully encompassing all the aspects of HIBD-induced motor impairments in CP. Although there are similarities in brain development between rodents and humans at postnatal week 1 and in late gestation, respectively, the rodents demonstrate the development of rapid motor coordination during postnatal weeks 1–2, which is equivalent to 1–2 years of age in humans (Cavarsan et al., 2019). For developing a better understanding of the neurophysiopathology of the brain, age-specific models across multiple species encompassing ischemic conditions should be employed (Mallard and Vexler, 2015). Notably, studies indicate an accelerated development of the central nervous system in rodents. Within a few days, they reach a developmental stage that is comparable to several months of human development, which imposes limitations in the investigation of functional nervous system development in rodents (Clowry et al., 2014). The findings of the current study were based on neuro-motor functional assessments, and they indicated a strong potential for motor recovery in rats.

Thus far, a significant challenge with most of the HIBD animal models has been their inability to completely replicate specific motor deficits that are observed in CP in humans. The focus of developing these animal models was to induce brain injuries that produced neuropathological changes similar to those seen in human CP. However, it has been observed that the HIBD animal models often fail to present a complete range of impairments in motor and posture control as observed in CP (Brandenburg et al., 2019). The neuro-motor functional experiments in the current study demonstrated a remarkable capacity for self-recovery in young rats. Nonetheless, HIBD animal models, which primarily include rodents, do not demonstrate a consistent replication of the severity of the central nervous system (CNS) damage and the resulting neurological and motor impairments. Notably, motor impairments in these models may be mild even with severe CNS damage. Furthermore, there are discrepancies in neuroanatomy and development between humans and rodents, which restricts the use of rodents as models for neuro-motor manifestations of HIBD in humans. Moreover, motor control involves signals from various brain regions, which necessitates the integration of multisensory information. The basal ganglia and the cerebellum influence posture and gait modulation in humans through connections with the cerebral cortex and the brainstem, respectively (Takano et al., 2020). The limitation of employing these animal models to infer HIBD-induced defects in humans arises from the relatively simplistic nature of brain injuries in animals, which cannot fully replicate the intricacies of human etiology and disease manifestation. Nevertheless, rodent models offer inherent advantages, which include affordability, accessibility, well-established techniques, ease of handling, and feasibility of transgenic experiments; hence, they continue to be extensively utilized for the investigation of the impact of brain damage on neuro-motor functions. Consequently, they remain indispensable to research (Silbereis et al., 2010).

Studies have demonstrated that the ligation of the carotid artery induces a slight reduction in cerebral blood flow on both the ipsilateral and contralateral hemispheres of the brain by approximately 20 and 5%, respectively (Buckley et al., 2015). Diminished local cerebral blood flow is a crucial determinant for assessing the magnitude of damage to the immature brain tissue (Ohshima et al., 2012). In the investigation, a rapid decline was observed in the model group, followed by gradual recovery of cerebral blood perfusion on the ipsilateral side. By day 7 post-modeling, no notable difference was observed in the mean perfusion of cerebral blood flow between the model and the sham group on the ipsilateral side. This suggests that the cerebral blood flow in the rats had been replenished, which could be attributed to extensive collateral circulation as well as robust vascular regeneration and remodeling capabilities that are observed in the rat brain. Even after ligating the left carotid artery, the blood flow could be sustained through collateral circulation and alternative pathways.

Downregulation of CD31 serves as an indicator of vascular endothelial cell damage. Blood flow decelerates following early ischemia, which leads to adherence of neutrophils to the ischemic endothelial cells, thereby initiating an acute inflammatory response (Han et al., 2023). On the first day post-modeling, the CD31-positive area ratio exhibited a significant reduction in the model group as compared to the sham group. However, this ratio gradually increased over time. By day 7, there was no significant difference as compared to the sham group, which could be attributed to the robust self-repair capability of the vascular endothelium of rats and the presence of ample cerebral collateral circulation that fosters angiogenesis. Furthermore, the restoration of cerebral blood flow exhibits a proportional correlation with the expression of CD31-labeled microvasculature, thereby underscoring the process of self-repair of damaged vascular endothelial tissue in the brain. Studies have demonstrated that CD31 is a pivotal pro-angiogenic factor that is involved in blood vessel formation. Hypoxia triggers CD31 expression, which aligns with enhanced angiogenesis that is observed after ischemic events. In summary, the initial downregulation of CD31 post-modeling and its subsequent increase signifies the dynamic transformations within the vascular endothelial cells and their repair mechanisms. Furthermore, CD31 expression is closely associated with the restoration of cerebral blood flow and the promotion of angiogenesis, which emphasizes the significance of vascular endothelial repair mechanisms in the alleviation of tissue damage following ischemic events (Zhang et al., 2021).

On the first day after HIBD, a significant decrease in the density of NeuN-labeled neurons is observed. It is well-acknowledged that the activation of microglia in diseased brains can induce the secretion of cytotoxic molecules, which poses a potential harm to the neighboring cells, including neurons (Lindhout et al., 2021). The affected neurons demonstrate degeneration, which is characterized by a reduction in the body size of the cells, nuclear deformation, and nuclear condensation. By day 4, certain neuronal cells that are necrotic undergo swelling, which leads to an increase in the cell volume. On day 7, fluorescent imaging demonstrates irregular staining of certain neurons, which is indicative of neuronal damage. During this period, the rat pups are in an actively developing neural state and exhibit robust regenerative capabilities for the degenerated neurons. In the current study, a pronounced decrease in the number of cortical neurons as well as neuronal damage, including necrosis, was observed on the first day after HIBD in rats. We attribute this outcome to the neurotoxins released by activated microglia. Furthermore, neurotoxicity induced by activated microglia also adversely affected the motor function of rats.

Central nervous system injury and the secreted factors can trigger astrocyte activation. The morphological changes observed in the activated astrocytes are a response to CNS injury. This response aims to maintain the neural environment homeostasis, repair the damaged tissues, and support the survival and recovery of the neurons (Konishi et al., 2020). GFAP, which is a type III intermediate filament protein, is predominantly present in the astrocytes of the central nervous system. GFAP plays a role in the formation of the cellular cytoskeleton to provide tensile strength. Moreover, it is commonly utilized as a marker for astrocytes (Stavale et al., 2013; Hol and Pekny, 2015). Increased GFAP expression serves as an important characteristic of reactive astrocytes that are activated in pathological processes such as hypoxia, infection, and trauma (Escartin et al., 2019). The current research demonstrated that following HI injury, the astrocytes undergo activation and continuous proliferation in the motor cortex. These astrocytes exhibit enlarged and thickened processes, increased cell body size, and migration to the injured cortex, which collectively assists in the restoration of the microenvironmental stability of the brain.

The microglia are immune cells that resemble macrophages, and they serve as the primary active immune defense cells against various stimuli. An increase in the number of microglia is characteristic of nearly all the pathological conditions of the central nervous system (Guo and Zhu, 2021). The microglia originate external to the central nervous system; however, they reside within the brain parenchyma (Prinz et al., 2019). Iba-1 is widely used as a marker for microglia, and it plays a crucial role in the maintenance of homeostasis and protection of the central nervous system under various pathological conditions (Korzhevskii and Kirik, 2016; Lituma et al., 2021).

Regarding HIBD, the role of microglia is complex and dual in nature. On one hand, the microglia contribute to neurotoxicity by secreting pro-inflammatory factors that exacerbate brain damage. Conversely, it also engulfs cellular debris and promotes brain tissue repair (Biber et al., 2007). Further, the microglia serve as immediate sensors of pathological changes in the brain, thereby playing a crucial role as the primary and major neuroglial cells that are involved in HIBD response during the neonatal period (Lv et al., 2020). In the rat cerebral cortex, microglia undergo a developmental process within the first 5 days after birth. They are termed as “primitive microglia,” and are characterized by the growth of numerous branches. Over a week, they gradually mature into “resting microglia,” with a distinctive branching morphology, and the number of microglia progressively increases each day. The morphological maturity of the “resting microglia” is first observed in the cells within the hippocampal structure and hypothalamus on day 5, followed by the cerebral cortex on day 7, and finally in the white matter on day 17 (Murabe and Sano, 1982). During cerebral hypoxic–ischemic events, the activation of microglia leads to the upregulation of the expression of Iba-1. The current research demonstrated that microglia are upregulated as early as the first day after HIBD. Activated microglia respond to hypoxia and ischemia by undergoing proliferation and migrating to the motor cortex. Furthermore, they experience cellular enlargement, shorter processes, and transition from a resting state to a highly branched morphology, thereby enabling an immediate response to HIBD. Inflammatory reactions can be regarded as necessary physiological responses. When the brain homeostasis is disrupted, the microglia initiate an acute inflammatory process that functions as a defense and repair mechanism (Garcia-Revilla et al., 2019; Rodriguez-Gomez et al., 2020).

Arg-1 is expressed in various cell types, particularly in immune cells, liver, kidney, and certain tumor cells. In humans, peripheral blood monocytes exhibit high expression of Arg-1, especially in neutrophils, and Arg-1 release occurs during the inflammatory process (Xu Z. et al., 2020; Denorme et al., 2022). Studies have demonstrated that the upregulation of Arg-1 in the ischemic hemisphere is primarily observed in brain-infiltrating macrophages, while the microglia do not express Arg-1 (Zarruk et al., 2018). Experimental evidence also suggests that the expression of Arg-1 increases during normal neurodevelopment of neonatal rats from P9 to P17, and it is primarily localized to microglia. Morphological changes and accumulation of Arg-1-labeled microglia have been observed as early as 4 h after injury (Mike et al., 2021). Previous studies indicate that Arg-1 is primarily associated with the function of “repair-promoting” microglia, and it is commonly used as a marker. Further, it plays a role in arginine metabolism (Tang and Le, 2016; Lisi et al., 2017; Krystofova et al., 2018). By releasing anti-inflammatory cytokines and synthesizing endogenous neurotrophic factors in the brain, Arg-1 can regulate the inflammatory process and protect neurons from adverse environmental effects, thereby exhibiting neuroprotective effects (Cherry et al., 2015). In general, the expression of Arg-1 in the brain tissue is low. However, after cerebral ischemia, a subset of microglia and macrophages upregulates Arg-1 expression (Li et al., 2021). In the current study, an increased presence of Arg-1-positive cells suggested their potential involvement in the regulation of anti-inflammatory processes post-HIBD. The upregulation of Arg-1 in the microglia may play a role in regulating nitrogen metabolism, neurotransmitter production and release, and modulating inflammatory responses. The activity of Arg-1 interacts with other metabolic enzymes to regulate microglial function and inflammatory levels. To examine the relationship between Iba-1 and Arg-1, fluorescence co-localization analysis was performed, which demonstrated a Pearson’s *R* value of 0, indicating no co-localization between the two. Interestingly, the fluorescence images show that Arg-1 is distributed around the microglia. Further investigation is required to elucidate the underlying reasons for this observation.

We observed a noteworthy increase of CD68-labeled microglia in the cerebral cortex of rats with HIBD, suggesting their activation and heightened phagocytic activity. As part of its protective function in the central nervous system, the microglia play a crucial role in facilitating neurogenesis and suppressing inflammation during hypoxic–ischemic conditions. These cells are primarily responsible for the clearance of deceased neurons during inflammatory responses; moreover, they contribute to the restoration of neuronal function.

Microglia and astrocytes are the main cell types that respond to various types of brain injuries. However, their functions are increasingly recognized as complex, frequently interacting, and sometimes even synergistic. They often play a beneficial role in optimizing central nervous system function (Liddelow and Barres, 2017). The interaction between microglia, neurons, and astrocytes plays a crucial role in maintaining endogenous plasticity to promote brain ischemic repair and functional outcomes (Sandvig et al., 2018). The phagocytic function of microglia involves the clearance of pathogens and dead cells, inflammation inhibition, and brain repair promotion, which contributes to the restoration of tissue homeostasis. The dynamic interplay between microglia and neurons as well as other types of glial cells is vital for a healthy brain. Besides acting as the immune guardians and preventing infection and inflammation, these cells also aid in maintaining brain homeostasis throughout, from early development to adulthood (Ginhoux and Garel, 2018; Gogoleva et al., 2019; Tan et al., 2020).

Glial cells, which include astrocytes, microglia, and oligodendrocytes, are essential components of the central nervous system. In normal physiological conditions, they actively participate in the development of the central nervous system (Xu S. et al., 2020). The astrocytes specifically contribute to the formation and maintenance of the blood–brain barrier, safeguarding the brain against pathogens and drugs (Lien et al., 2019; Byun et al., 2020). Additionally, the astrocytes secrete neurotrophic factors and circulate neurotransmitters, thereby ensuring homeostasis in the central nervous system. They also play a crucial role in regulating synaptic plasticity to support the normal physiological function of the neurons. Traditionally, NVU did not incorporate oligodendrocytes. However, recent investigations have shed light on the important role of oligodendrocytes within the NVU. These cells are responsible for the formation and maintenance of myelin sheaths, thereby enabling the efficient conduction of electrical impulses and the preservation of axon integrity (Lee and Hur, 2020).

In summary, this study aimed to investigate the effects of neonatal HIBD on neuro-motor function, cerebral blood flow, and neurovascular units in rats. The findings provide valuable insights about the impact of HIBD on the motor cortex. The experimental results in the current study serve as a reference for understanding the early response mechanisms to HIBD and offer potential therapeutic strategies from the perspective of the NVU to mitigate the development of cerebral palsy caused by HIBD. During the investigation of early changes in HIBD, a sustained upregulation of glial cells was observed, without them reaching a turning point. Additionally, there were indications of recovery in cerebral blood flow, microvasculature, and neuro-motor function. To assess the long-term effects of HIBD on NVU, the observation period would need to be extended in future studies.

It is important to note that the current study focused solely on glial cells and did not explore other components of the neurovascular unit, such as pericytes and oligodendrocytes. Furthermore, there is a lack of research on the interplay between the neurovascular units. To address these knowledge gaps, further studies will be conducted.

## Data availability statement

The raw data supporting the conclusions of this article will be made available by the authors, without undue reservation.

## Ethics statement

The animal study was approved by the Animal Ethics Committee of the Institute of Basic Theory of Traditional Chinese Medicine, China Academy of Chinese Medical Sciences. The study was conducted in accordance with the local legislation and institutional requirements.

## Author contributions

YM designed and conducted the experiments, prepared figures, analyzed the data, and wrote, edited, and proofread the manuscript. YZ organized and analyzed the data, and wrote, edited, and proofread the manuscript. LH, YJ, TZ, ZZ, and JT assisted in the implementation of animal experiments. GL polished the manuscript. XM conceived and supervised the entire project. All authors contributed to the article and approved the submitted version.
